# *Leishmania V. braziliensis* infection in asymptomatic domestic animals within an endemic region in the Northeast of Brazil

**DOI:** 10.1590/0037-8682-0600-2021

**Published:** 2022-08-12

**Authors:** Claudio Júlio da Silva, Karina Patricia Baracho Lima, Juliana Figueirêdo da Costa Lima Suassuna Monteiro, Andréa Karla Sales Ferreira da Silva, Fernando José da Silva, Allana Maria de Souza Pereira, Valéria Pereira Hernandes, Elis Dionísio da Silva, Cláudia Sofia de Assunção Gonçalves e Silva, Sinval Pinto Brandão, Maria Edileuza Felinto de Brito

**Affiliations:** 1 Fundação Oswaldo Cruz, Instituto Aggeu Magalhães, Departamento de Imunologia, Laboratório de Imunoparasitologia, Recife, PE, Brasil.; 2 Núcleo de Vigilância em Saúde de Moreno, Moreno, PE, Brasil.; 3 Universidade Fernando Pessoa, Faculdade de Ciência e Tecnologia, Porto, Portugal.; 4 Universidade Fernando Pessoa, Centro de Investigação em Biomedicina, Unidade de Investigação em Energia, Ambiente e Saúde, Porto, Portugal.

**Keywords:** American cutaneous leishmaniasis, Reservoirs. Molecular Diagnostic

## Abstract

**Background:**

American cutaneous leishmaniasis is a commonly neglected, vector-borne tropical parasitic disease that is a major public health concern in Brazil. *Leishmania (Viannia) braziliensis* is the main species associated with the disease. Accurate diagnosis is based on epidemiological surveillance, clinical assessment, and laboratory testing. *Leishmania (V.) braziliensis* has been detected in several wild and synanthropic mammals. Their epidemiological role has not been entirely elucidated. This study aimed to assess potential *L. braziliensis* infections in asymptomatic domestic animals, by molecular and serological testing in endemic areas, in the metropolitan region of Recife.

**Methods:**

Blood samples and conjunctival fluids were collected from 232 animals (canids, felids, equines, and caprines) for the detection of *L. braziliensis* using molecular tests (conventional and real-time polymerase chain reaction [PCR and qPCR]). For immunological detection, blood samples from 115 dogs were assessed using enzyme-linked immunosorbent assay.

**Results:**

Real-time quantitative PCR showed positive results for blood and conjunctival samples in all investigated species. The results of the blood and conjunctival samples were 68.2% and 26.9% in *Canis familiaris*, 100% and 41.7% in *Felis catus*, 77.3% and 30.8% in *Equus caballus*/*Equus asinus*, and 50% and 33.3% in *Capra hircus* samples, respectively.

**Conclusions:**

Results from this study adds valuable information to our understanding of the role of asymptomatic domestic animals, *L. braziliensis* life cycle, and American cutaneous leishmaniasis in Northeast Brazil.

## INTRODUCTION

Leishmaniasis is a vector-borne parasitic disease that is a global public health concern. The parasite *Leishmania spp* is transmitted to humans via the bite of infected females *Lutzomyia spp.* sandflies^1^. It encompasses a wide spectrum of clinical tropical diseases depending on different variables, such as the parasite species, vectors, reservoir hosts, and ecosystems, among others^2^.

American cutaneous leishmaniasis (ACL) is characterized by cutaneous impairment and eventual mucosal involvement. Brazil is one of the top five countries with the highest incidence of ACL worldwide^3^ ^,^ ^4^. ACL displays specific transmission patterns in Brazil, due to the variety of vectors and reservoir hosts^5^ ^,^ ^6^. Domestic and wild animals such as canines (*Canis familiaris)*, felines (*Felis catus)*, horses (*Equus caballus)*, donkeys (*Equus asinus),* and rodents (*Necromys lasirus, Nectomys squamipes, Rattus rattus*) are considered the main reservoir hosts of the disease^7^ ^,^ ^8^.

The state of Pernambuco, Brazil, has ACL cases predominant in Recife and its metropolitan region (Pernambuco Forest zone)^7^. It should be noted that human cases are generally believed to be under-reported. Between 2003 and 2018, 412 human cases were reported in the city of Moreno, Pernambuco, an endemic region for ACL. In 2018, 14 cases were reported during the sample collection in this region^9^ ^,^ ^10^.

The onset of clinical manifestations in humans presents as small papules that may evolve into multiple skin ulcers^11^ ^,^ ^12^. Infected animals may develop lesions owing to diverse intrinsic factors. The disease severity is related to the host’s immune response. However, asymptomatic cases of infection often occur^13^.

The accuracy of available diagnostic tools is enhanced when combined with precise analyses, clinical evaluation, and other correlated laboratory findings^14^. This study aimed to assess leishmaniasis infection in asymptomatic domestic animals, using molecular and immunological tools for early detection. Infection detection in asymptomatic animals will provide knowledge of potential hosts for *Leishmania spp.* and improve local epidemiological surveillance.

## METHODS

### Population and biological samples

**FIGURE 1:**
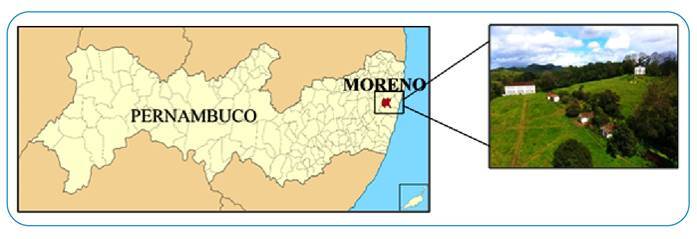
Map of Moreno, a municipality in the Recife metropolitan zone. The red spot on the map depicts the municipality of Moreno, from where the study samples were obtained.

Samples were collected from dogs between January 2017 and January 2018. All biological samples were from Moreno city (endemic area for cutaneous leishmaniasis), located 28 km from Recife, the capital of Pernambuco, with a rural population of 6,499, and from one of these two regions: Engenho Jardim (approximately 304 inhabitants) and Engenho Cumarú (approximately 441 inhabitants)^15^, where both animals and humans live (Figure 1). Each tutor was interviewed and completed a questionnaire to provide epidemiological data.

At the time of sample collection, a veterinarian clinically examined each domestic animal. All signs and symptoms were noted in the animal’s clinical records. Based on this examination, all animals were classified as symptomatic or not, using the following criteria for case definition: presence of active skin lesions, living in an endemic area (Moreno region), and with a laboratorial positive test for leishmaniasis. Animals with no active skin lesions who lived in the studied region and had a positive laboratory test for leishmaniasis were classified as asymptomatic. All animals with negative diagnostic tests were classified as those without leishmaniasis.

Convenience sampling was adopted, and biological material from domestic animals was collected from the two endemic areas for ACL. A total of 272 animals were selected for the study (212 *Canis familiaris*, 33 *Equus a. asinus/Equus f. caballus*, 21 *Felis catus*, and six *Capra a. hircus*), but some animals were unable to participate because of one or more of the following selection criteria: very aggressive animals; animals were not at home at the time of collection; or little biological material was collected for laboratory tests.

In total, 188 dogs (*Canis familiaris*) were assessed, and peripheral blood and conjunctival fluid samples were collected. In addition, samples from nine cats (*Felis catus*), 29 horses (*Equus f. caballus/ Equus a. asinus*), and six goats (*Capra a. hircus*) were collected to investigate leishmaniasis at IAM/FIOCRUZ-PE. A total of 232 samples from asymptomatic animals were processed by the Immunoparasitology Laboratory of IAM/FIOCRUZ-PE.

### Ethical aspects

Informed consent form was signed by every tutor. The present study was approved and safeguarded by the Committee on Animal Research and Ethics of IAM/FIOCRUZ-PE (protocol number 115/2017).

### Methods for detection of *L. (V.) braziliensis* in asymptomatic animals

The study used two different approaches: molecular and immunological. Real-time quantitative polymerase chain reaction (qPCR) was used as the molecular method, which was based on the protocol described by Paiva *et al*.^16^. Enzyme-linked immunosorbent assay (ELISA) was used as the immunological tool.

## MOLECULAR TESTING

DNA samples from blood leukocytes and conjunctival swabs were processed using qPCR. This system uses kDNA as a detection target for the variable region of *L. (V.) braziliensis* minicircular kinetoplast, amplifying a 138pb fragment. All samples were tested twice with positive and negative controls. Conventional PCR was performed targeting parasite kDNA, equivalent to 750pb and in accordance with settings described^14^.

## PLASMA AND LEUKOCYTES ISOLATION

 Leukocytes and plasma from peripheral blood were separated by the concentration gradient technique using Ficoll-Hypaque, in agreement of referred protocol^16^. To facilitate communication, blood samples from animals were only denominated as leukocytes after separation into two phases: plasma and leukocytes. Only leukocytes were used for the molecular experiments. 

## DNA EXTRACTION

DNA was extracted from blood leukocytes and conjunctival swab samples using a Qiagen commercial kit (QIAmp® DNA and Blood) according to the supplier’s instructions. DNA concentration curves were used to *define Leishmania (V.) braziliensis* DNA detection sensibility. These curves used reference strains of *L. (V. braziliensis* IOC-566-MHOM/BR/75/M2903. The DNA was diluted to a concentration of 10-10ng/L to 1fg/μL. 

## ELISA

ELISA was performed according to protocol developed *in house*. The protocol was adjusted to use anti-dog IgG (gamma chain specific) conjugated to peroxidase and the soluble *L. (V.) braziliensis* antigen fraction.

### *L. (V.) braziliensis* promastigotes obtainment

Culture aliquots of *L (V.) braziliensis* promastigotes were obtained from the reference strain MHOM/BR/75/M2903 and cultured in Schneider’s medium until the exponential growth stage.

The parasites were then removed from the supernatant and washed three times with buffered saline solution containing 10% bovine fetal serum (BFS). The mixture was then centrifuged (4°C, 871 × g) for 10 min. The sediment was resuspended in 1% paraformaldehyde and incubated for 24 h at 4°C. Parasites were washed twice with 3% BFS after incubation. Finally, an aliquot was prepared, and parasite counting was performed inside the Neubauer chamber.

### Obtaining a soluble *L. (V.) braziliensis*

Promastigotes were transferred *in vitro* to sterile Falcon tubes and centrifuged at 400X g for 10 min with sequential washes (3x) in buffered saline solution pH 7.2 (1x). Finally, the pellet was resuspended in 750μL of mild lysis buffer solution and 250μL of protease inhibitor.

For cell lysis, the final content was transferred to 1,5mL microtubes and subjected to successive frosting and defrosting with liquid nitrogen and heated bathing at 36ºC, respectively. Nearly 20μL of the final solution was analyzed using optical microscopy for cell viability and other freezing/defrosting sessions, if necessary.

The resulting solution was centrifuged at 10, 000 g for 15 min. Soluble antigens were collected in the supernatant and then quantified using the Bradford protein assay (10μL of antigen suspension; 200μL of Bradford reagent and 790μL of deionized water), aliquoted in 1.5mL microtubes and frozen at -80°C thereafter.

### Statistical analysis

A database for recording the results was built using IBM SPSS Statistics 20.0 statistical software. Regarding disease prevalence within the studied population, *cutoff* calculations as well as tables and figures were built using the SPSS Program and GraphPad Prisma version 7.0 (GraphPad Prism Inc. San Diego, CA). All conclusions were made at the 5% significance level.

## RESULTS

Clinical evidence revealed 13.2% of domestic animals with skin lesions, 0.7% of them with lymphadenopathy. During the study, 14.6% of the animals had no laboratory confirmation of ACL, based on a survey conducted in two mills in the region. Among asymptomatic animals, the absence of lesions was clinically proven in 87.3% (unpublished data). 

Samples obtained from 232 asymptomatic domestic animals were divided into five different groups according to species: *Canis familiaris; Felis catus; Equus caballus; Equus asinus;* and *Capra hircus*. The analytical limit of detection (LOD) of DNA was determined using a reference DNA strain (IOC-566-MHOM/BR/75/M2903) of *L. (V). braziliensis* in the conventional PCR was 10fg. Real-time quantitative PCR (qPCR), which accounted for 98% efficiency rates, had a minimal LOD was 1fg of *L. (V). braziliensis* DNA.

**FIGURE 2:**
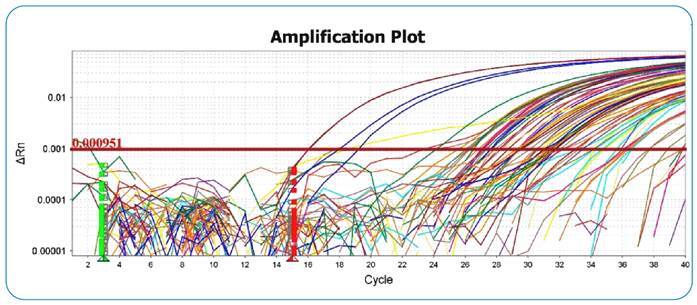
qPCR amplification of *L. (V.) braziliensis* DNA in leukocytes. Graph with the amplification curve of a qPCR experiment with a standard curve (1ng of DNA to 1fg of DNA) and leukocyte samples from domestic animals. The reaction had an efficiency higher than 93%.

**FIGURE 3:**
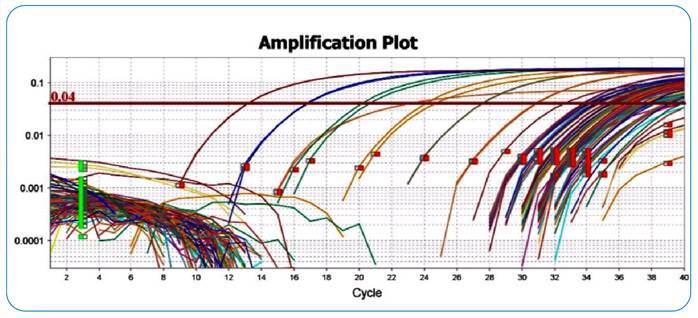
qPCR amplification of DNA from *L. (V.) braziliensis* in a conjunctival swab. Graph with the amplification curve of a qPCR experiment with a standard curve (1ng of DNA to 1fg of DNA) and conjunctival swab samples from domestic animals. The reaction had an efficiency higher than 94.5%.

For leukocyte samples processed by qPCR, the peak of the dissociated curve used, according to Paiva et al.^15^, was in the range between 79°C and 82°C, with a specific peak for *L.* (*V.*) *braziliensis* at 81.03ºC. To illustrate the results of the qPCR analysis, Figure 2 shows a graph of an amplification curve with samples of leukocytes from domestic animals. The positive results obtained from qPCR (leukocyte samples and conjunctival swabs) are described in Supplementary Table 1. The amplification graph for the conjunctival swab samples is shown in Figure 3.

The average quantity of *L.* (*V.*) *braziliensis* DNA within leukocyte samples and conjunctival *swabs* was 40.1fg and 35.1fg, respectively. Quantification of DNA in leukocyte samples by species was as follows: *Canis familiaris*:50.78fg, *Felis catus*:3.43fg, *E. caballus*/*E. asinus*:17.78fg, *Capra hircus*:29.36fg. For conjunctival swab: *Canis familiaris:* 34.87fg, *Felis catus*:19.39fg *E. caballus*/*E. asinus*:47.51fg *Capra hircus*:12fg.

**FIGURE 4:**
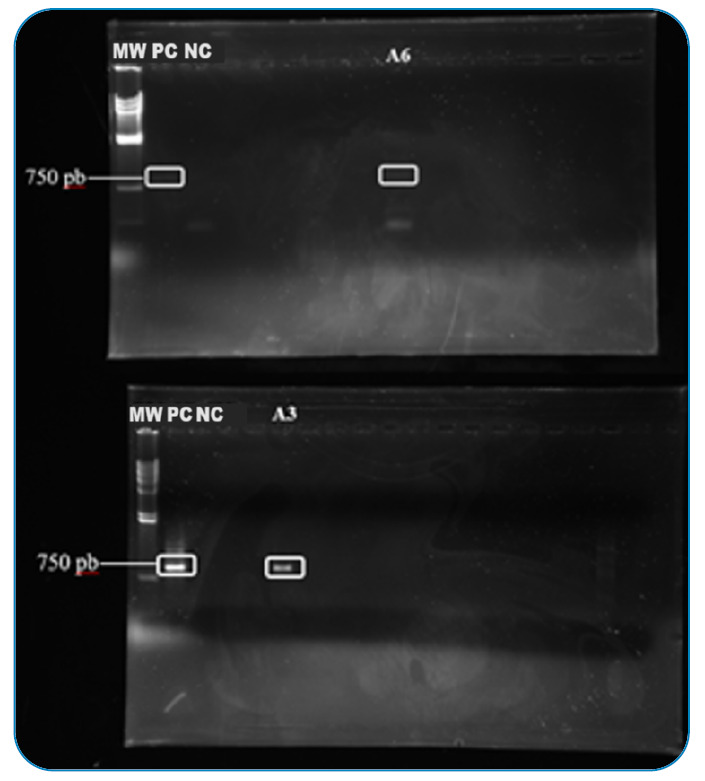
Agarose gel amplification of *L. (V.) braziliensis* DNA in leukocytes. **MW:** molecular weight; **PC:** positive control; **NC:** Negative control; **A6:** sample 6; **A3:** Sample.

For molecular reactions, conventional PCR and qPCR were compared for their positivity for each species and clinical sample type. Regarding conventional PCR of leukocytes, two samples obtained 750pb amplification, animals positive for *L. (Viannia)*, and all others (including leukocytes and conjunctival swab) were negative for all species. Both positive animals on conventional PCR belonged to *Canis familiaris* species, accounting for a percentage frequency of 1.1%. On qPCR, more samples (leukocytes and conjunctival swab) were positive for *L. (V.) braziliensis* ranging positivity (by species and clinical sample type) was from approximately 27% to more than 77% by species and clinical sample type. All the details are shown in Supplementary Table 1 and Figure 4.

Comparing the positivity percentage of conventional PCR and qPCR, on leukocytes on *C. familiaris*, positivity on qPCR was 67.14% (CI = 59.66-73.42) higher than that on conventional PCR, with *p* < 0.0001. The chi-square test result was 186.68. 

## ELISA

**FIGURE 5:**
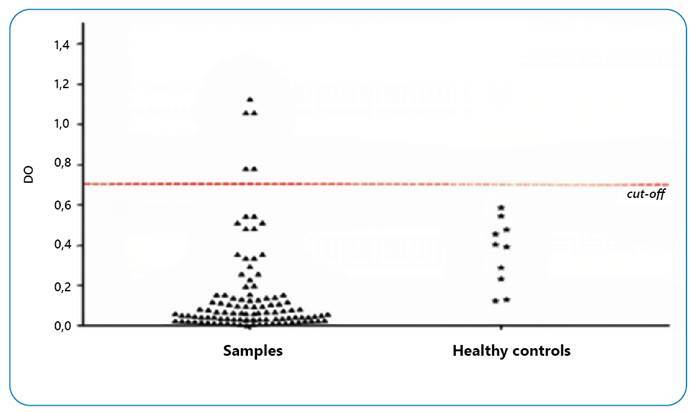
Infection prevalence with *L. (V.) braziliensis* in *Canis familiaris.* Detection threshold (*Cutoff*): 0.689.

ELISA was performed to determine *L. braziliensis* prevalence within the dog population (n = 115). Ten healthy animals were used as the negative controls. On average, 4.35% of the dogs tested positive using this method (Figure 5).

## DISCUSSION

ACL is an infectious-parasitic disease that has an ecoepidemiology to be able to establish different transmission cycles, including varieties of reservoirs and vectors, depending on each region. The role of domestic animals in the epidemiological cycle of ACL in the Americas has been described for years^17^ ^,^ ^18^. Approximately 21.3% of asymptomatic animals can infect sand flies with *Leishmania* spp^19^. In humans, population survey studies in Engenhos Jardim and Carnijó showed a prevalence of 22.4% and in another mill in the region, Engenho Pinto, showed a prevalence of 30%. All three cited locations were in the same municipality of Moreno-PE^20^ ^,^ ^21^.

Infection by *Leishmania* spp. in domestic animals can be asymptomatic, oligosymptomatic, or symptomatic. Approximately 60% of dogs are asymptomatic, and clinical signs and symptoms can take months or years to appear after the infection. When these symptoms develop, they first occur as skin lesions and evolve into systemic form^5^ ^,^ ^20^. To develop a highly sensitive detection system for asymptomatic *Leishmania spp*. infections in domestic animals, two different systems were tested. Molecular systems based on PCR were tested by conventional PCR and qPCR. Given their high detection sensitivity, kDNA targets have been used in molecular testing^22^. Regarding blood samples and conjunctival swab samples, Conventional PCR yielded only two positive results for blood and conjunctival swab samples. When compared to qPCR, conventional PCR on leukocytes from *C. familiaris* had a positivity percentage that was almost 70% smaller, with statistical significance. This may be because qPCR is more sensitive to DNA detection than conventional PCR^23^, and to protocol variations among different systems. Several factors influence DNA detection in a molecular testing system, such as extraction methods, initial *primers* selection, clinical sample types, and infection period of each animal, and the latter is directly related to parasite load. Biological samples and specimen collection methods are considered important factors for obtain optimal results^15^.

The qPCR is considered a fast, specific, and sensitive technique for parasite detection^24^ ^,^ ^25^ ^,^ ^26^ ^,^ ^27^. It is widely used for parasite DNA detection in diverse biological samples, including peripheral blood samples^28^. Previous studies^29^ ^,^ ^30^ have shown the possibility of detecting the presence of the parasite through this type of sample (eye and saliva swabs) using molecular techniques. The results were similar to those obtained from skin biopsy. The study population consisted of asymptomatic animals with no obvious lesions, justifying the use of less-invasive samples.

It is well-known that amastigotes of *Leishmania spp.* are preferentially encountered within polymorphonuclear cells (leukocytes), and thus, this sample was chosen for testing. It should be noted that the results of this study revealed positivity among leukocyte samples when using qPCR and, therefore, were consistent with amastigote behaviour^26^. Excluding qPCR for *Capra. a. hircus*, which was not statistically significant in other species, leukocytes presented higher positivity than conjunctival swabs. However, the fact that conjunctival swab samples showed less positivity when compared to leukocyte samples might indicate that it is not a site of parasitic tropism. However, conjunctival samples are less invasive and easier to collect in animals, whether in rural areas or ambulatories^31^. Additionally, qPCR does not necessarily detect intact or viable parasites. It can detect fragments of target DNA from *L. (V.) braziliensis,* which means that DNA can freely circulate between fluid and tissues’ host^32^.

Among the species of animals studied, the absolute DNA concentration was higher in leukocytes obtained from dog samples. Cats had the lowest average DNA concentration in blood samples among the species (less than 5fg). Feline leishmaniasis has been identified in tropical and subtropical regions worldwide. However, the role of felines as reservoir hosts is unclear. In Brazil, cats are potential reservoir hosts for *L. (V.) braziliensis* ^32^ ^,^ ^33^ ^,^ ^34^.

The evaluation of existing transmission cycles involving domestic animals is necessary to prove parasite transmission to sandfly vectors using infectiousness assays (xenodiagnoses)^32^ ^,^ ^33^. Based on the results presented, it could be assumed that all investigated animal species can be infected by *L. (V.) braziliensis* when they live in endemic regions. Further studies are necessary to prove the real participation of each in the maintenance of the transmission cycle of the ACL.

ELISA for antibody detection showed positive results in five dogs, accounting for 4.34% of the prevalence (5/115). This technique assesses the presence of pathogens in the blood plasma. According to the joint technical standard No. 01/2011-CGDT-CGLAB/DEVI/SVS/MS, the first-choice serological diagnosis for dogs recommended by the Brazilian Ministry of Health is the rapid immunochromatographic test denominated “TR DPP® Bio-Manguinhos,” which is used as a screening exam, while the indirect ELISA test is used as a confirmatory test^35^.

Despite being a definite serological diagnostic test for dogs, ELISA has technical limitations related to the difficulty in distinguishing between recent and previous infections, aside from cross-reactions. Surface antigens as well as antigens derived from protozoan cytoskeleton microtubules are common to species of the *Trypanosomatidae* family, which explains the occurrence of cross reaction among them^36^ ^,^ ^37^.

ELISA’s specificity may decrease when applied to epidemiological studies owing to the occurrence of nonspecific reactions^33^. Conventional PCR and qPCR are optimal diagnostic tools with high sensitivity for the detection of serum-negative dogs^38^ ^,^ ^39^. Moreover, both techniques allow efficient and accurate detection of parasite DNA, confirming diagnosis in animals. In addition, PCR systems are useful, especially in cases where serological investigations are inconclusive. When applied together with serological examinations, molecular systems may contribute to the evaluation of the extent of *Leishmania spp.* Infections^40^. 

In conclusion, this study represents a contribution to ACL epidemiology when approaching the assessment of asymptomatic domestic animals over an endemic area with robust sampling of diverse animal species, since it is well-known that most described studies predominantly focus on canine visceral leishmaniasis. The parasite load in the animals in this study demonstrated that even asymptomatic animals had contact with *L. (V.) braziliensis* and had circulating parasitic DNA. These findings are consistent with the hypothesis that asymptomatic domestic animals are reservoir hosts and sources of infection for nearby mammals and human populations. Considering these facts, new perspectives and a better understanding of the larger transmission cycle within the Recife Metropolitan Region are necessary.

## SUPPLEMENTARY MATERIAL

**SUPPLEMENTARY TABLE1:** Positivity of clinical samples (conjunctival swab and blood/leukocytes) on conventional PCR and qPCR for each animal species and comparison of clinical type on qPCR.

Animals by specie (n=232)	Conventional PCR		qPCR				
	**Conjunctival *swab***	Leukocytes	**Conjunctival *swab***	Leukocytes	Chi-square	Difference (CI%)	Significancy level (p)
***Canis familiaris* (n=188)**	0%	1,06%	26,9%	68,2%	64.11	41.3	**<0.001**
	(0/188)	(2/188)	(35/188)	(120/188)		(31.6-49.8)	
***64 Felis catus* (n=9)**	0%	0%	41,7%	100%	7	58.3	**0.008**
	(0/9)	(0/9)	(05/09)	(09/09)		(16.2-82.9)	
***Equus f. caballus / Equus a. asinus* (n=29)**	0%	0%	30,8%	77,3%	12.4	46.5	**<0.001**
	(0/29)	(0/29)	(04/29)	(17/29)		(21-64.4)	
***Capra a. hircus* (n=6)**	0%	0%	33,3%	50%	0.3	16.7	**0.574**
	(0/6)	(0/6)	(01/06)	(03/06)		(-31.5-55.9)	
